# Financial scarcity, psychological well-being and perceptions: an evaluation of the Nigerian currency redesign policy outcomes

**DOI:** 10.1186/s12889-024-18603-w

**Published:** 2024-04-25

**Authors:** Judith Ifunanya Ani, Vincent O. Ajayi-Ojo, Kezia Batisai

**Affiliations:** 1 Faculty of Humanities, University of Johannesburg, Johannesburg, South Africa; 2 LAPO Institute for Microfinance and Management Studies, Benin City, Nigeria

**Keywords:** Naira Redesign Policy, Nigeria, Psychological Well-bei, Psychological Inventory of Financial Scarcity, Cash Crunch

## Abstract

**Background:**

The relationship between insufficient financial resources and psychological health has been extensively studied and established in various contexts. However, there remains uncertainty regarding the potential impact of the Nigerian naira currency redesign policy on the psychological well-being of Nigerians. This policy, which aimed to demonetize the economy and promote economic stability, involved changes to the physical appearance of some naira denominations (200, 500 and 1000). Understanding the effects of this policy on psychological health is essential for evaluating its overall societal impact and identifying potential areas for improvement in future currency redesign initiatives.

**Methods:**

The study is a cross-sectional mixed-methods study involving 2237 respondents across the six geopolitical zones of Nigeria. Utilizing the simple random, snowball and convenience sampling technique, social media platforms (Facebook and WhatsApp) were used to recruit respondents. Variables were analyzed at descriptive and inferential levels. The qualitative component comprised seven (7) in-depth interviews with participants across the geo-political zones.

**Results:**

The perceptions of respondents towards the policy were diverse across different demographic groups. It was widely perceived that the timing of the policy was inappropriate, considering the challenges faced in utilizing online payment platforms and the significant inaccessibility of cash. Furthermore, the analysis revealed that demographic variables played a role in explaining systematic variations in the experience of financial scarcity and its effect on psychological health during the cash crunch that ensued as a result of the Nigerian naira currency redesign policy.

**Conclusions:**

This study identified a significant association between the psychological inventory of financial scarcity and psychological well-being among residents in Nigeria during the cash crunch resulting from the Naira redesign policy. The findings suggest that the financial scarcity experienced by Nigerians due to the policy had a substantial impact on individuals’ psychological well-being. We recommend that a holistic approach be undertaken by policymakers to ensure that policy actions not only address economic objectives but also safeguard the mental health and overall well-being of the population.

## Background

Insufficient financial resources significantly impact people’s lives, extending beyond their financial condition. It not only has negative financial effects but also brings about financial stress and poor psychological well-being. The experience of financial scarcity increases stress levels and anxiety [1–3]. Moreover, it has the potential to create “poverty traps” characterized by excessive borrowing, discounting future benefits, and financial avoidance [4]. The impact of financial scarcity goes beyond the economic realm and directly affects psychological well-being. Mental health, an essential aspect of psychological well-being, encompasses more than just the absence of mental disorders or symptoms. It is a valuable resource that promotes overall well-being and productivity. Positive mental health involves individuals recognizing their abilities, effectively coping with everyday stresses, engaging in productive and meaningful work, and contributing to their communities [5]. However, economic shocks such as the demonetization of an economy have the potential to destabilize one’s overall psychological well-being including mental health, leading to detrimental impacts [6].

On October 26, 2022, the Central Bank of Nigeria (CBN) announced the sudden demonetization of the economy, which involved the redesign of the highest three Naira (₦) denominations (₦1,000, ₦500, and ₦200). Alongside this, the CBN implemented withdrawal policies that initially limited individuals to ₦100,000 per week for withdrawals from Automated Teller Machines (ATMs), Point of Sale (POS) terminals, and over-the-counter locations, while corporate organizations faced a withdrawal limit of ₦500,000, although this was reviewed upwards to ₦500,000 and ₦5,000,000 for individuals and corporate bodies respectively [7–10]. These currency redesign and withdrawal policies had significant and undeniable consequences in the country. One immediate impact was the scarcity of the Naira currency, leading to widespread hardship and a cash crisis with no immediate relief in sight. The scarcity of the new Naira notes also resulted in negative behaviours among individuals unable to access their money in banks, leading to acts of vandalism, including damaging bank ATMs and properties. This compelled several commercial banks to shut down operations.

The aftermath of the policy also had detrimental effects on the livelihoods of banking agents and Point of Sales (POS) operations in Nigeria [11]. A survey conducted by the Aggregating Officer of Mobile Money and Banking Agents in Nigeria (AMMBAN) highlighted the impact on mobile money and banking agents in Nigeria. The survey revealed that a staggering 80% of AMMBAN members were forced to shut down their operations due to the scarcity of Naira notes. This closure directly and adversely affected POS operators throughout the country, with some locations experiencing a drastic reduction in the number of operators from ten to just two [12].

Scarcity of cash, accompanied by economic decline and crisis, has been widely recognized as a contributing factor to various psychological well-being issues, including depression and suicide [13–16]. This link becomes evident when examining global financial crises, where a surge in suicide attempts and actual suicides were observed in countries implementing austerity measures, such as Greece, Ireland, and England [17–21].

These findings support earlier reports establishing a connection between financial downturn and increased suicide rates in various European countries [22]. Similarly, in an Indian survey examining the impact of the cash crunch on mental health, psychiatrists reported a significant number of patients suffering from mental stress following the demonetization of Rs 500 and Rs 1000 currency notes. Many of these patients experienced panic and anxiety attacks, depression, and suicidal thoughts [23]. Similarly, a study conducted in Bangalore, India, which examined customers’ perception towards demonetization, concluded that customers were negatively impacted during the demonetization period [24]. Furthermore, scholars argued in a related study that demonetization had disproportionately negative consequences on the mental health of the poor [25].

In a Spanish study [26], a significant increase in the proportion of patients with mood depression, generalized anxiety disorder, somatoform disorders, and alcohol-related disorders was found following an economic crisis. Additionally, the World Health Organization reported that the economic crisis of 2007 had secondary mental health effects, leading to increased suicide and alcohol-related death rates.

While there is extensive research on demonetization and psychological well-being, it is noteworthy that most of these studies have been conducted in advanced nations with only a limited number of studies conducted in less developed nations. In the context of Nigeria, there is a significant gap in the literature regarding demonetization and its impact on psychological well-being. It is important to note that previous cash redesign policies in Nigeria did not result in severe immediate cash scarcity as experienced under the current policy. Therefore, given the significance of psychological well-being, it becomes imperative to assess how the Nigerian currency redesign has led to financial stress and its subsequent effect on the psychological well-being of Nigerians. By investigating this relationship, we can gain a better understanding of the unique challenges faced by individuals in Nigeria during the demonetization process and inform future policies and interventions to mitigate the negative psychological consequences.

## Method

### Study design

The study was cross-sectional. It was a triangulation of methods that includes self-administered e-survey which the respondents completed anonymously and in-depth interviews conducted through telephone with participants across the geo-political zones.

### Study setting

The study was conducted across the six (6) geopolitical zones in Nigeria (North-east, North-west, North-central, South-east, South-south and South-west).

### Study population and inclusion criteria

The study targeted eligible Nigerians aged 18 years and above, resident in any of the geopolitical zones in Nigeria, for at least the past six months preceding the study, irrespective of gender, educational attainment, religion and socioeconomic background.

### Sampling and sample size

For the quantitative survey, a sample size of 2237 respondents was recruited across the six (6) geo-political zones in Nigeria using the simple random, convenience and snowball sampling technique. The researchers utilized e-survey using social media platforms (Facebook and WhatsApp) to recruit respondents and gather the survey data. Surveys offer several advantages, including access to a broad population, resulting in increased statistical power. They enable the collection of substantial amounts of data and provide the option to utilize validated models [27]. Social media, on the other hand, has been acknowledged for its wide reach and large number of subscribers [28], making it an increasingly primary source of data collection for social research [29, 30]. A questionnaire response link was generated by the researchers and the link shared on the aforementioned social media platforms. Preliminarily questions were included to ensure that only eligible respondents completed the questionnaire. These preliminaries questions were being a Nigerian, being 18 years and above, and having resided in Nigeria in the last 6 months that preceded the study. Respondents who were not Nigerians, who were below 18 years and/or have not resided in the country in the past 6 months that preceded the study, were prompted to discontinue the interview. The researchers, guided by the principles of theoretical saturation, conducted seven (7) in-depth interviews across the geo-political zones to elicit participants’ views on their perception of the redesign policy, its timing, and preparedness of country for a cashless economy, inclusivity and outcomes of the policy on psychological health of Nigerians. These were content analyzed.

### Data collection and analysis

Prior to initiating data collection, the authors conducted a pilot study. This preliminary investigation was conducted with the recognition of the pivotal role that pilot studies play, as emphasized by scholars [31]. The pilot study had several specific objectives. Firstly, it sought to evaluate the feasibility of the study and identify any potential weaknesses in the research design. Secondly, it aimed to assess the effectiveness of the study instrument in eliciting the intended information, while also scrutinizing the comprehensibility of the format. Additionally, the pilot study aimed to determine the appropriateness of the selected validated tools for the specific target population. It also examined the suitability of the data collection methods (e-survey using social media platforms) and lastly, assessed the time required for participants to complete the questionnaire and measuring participants’ willingness to actively participate in the study. The pilot demonstrated that the study was feasible and the method suitable. It also demonstrated that the instrument was appropriate and comprehensible, and that respondents were willing to complete the instrument. Another outcome of the pilot was the need to incorporate a qualitative aspect where participants expressed their perception of the policy and its effects. This helped generate a more robust and insightful data.

Quantitative data was collected with both open and closed-ended questionnaires. The questionnaire was designed in English language and took an average of 10 min to complete. Respondents were assured of confidentiality. Voluntariness, benefits, non-malfeasance and withdrawals were explicitly explained to respondents. The survey tool contained questions on socio-demographic characteristics, naira note redesign perception, Psychological Inventory of Financial Scarcity (PIFS), financial problems confronted with, and psychological well-being.

Psychological Inventory of Financial Scarcity (PFIS) assessed financial scarcity experienced by people [32]. PFIS has four components that measure aspects of people’s experiences. The first component elicits information on money scarcity, that is, insufficient financial resources. The second relates to the inability of one to have control over insufficient financial resources (lack of control over the financial situation). The third deals with “financial rumination” which describes worry and anxiety over the inability to solve problems and being uncertain about the financial future (financial rumination and worry), and the fourth is short-term focus. The instrument is both valid and reliable with a Cronbach Alpha(*α*) of 0.92. The instrument is construed on a 5-item scale ranging from totally disagree to totally agree. Higher scores indicate more financial stress.

We also included measures of financial problems with psychological well-being. This is because financial scarcity causes financial problems which affect the psychological well-being of an individual [33–36]. This is also on the assumption that financial scarcity leads to financial problems which results in certain psychological outcomes. Measures of financial problems variables were adapted. These included: not being able to make ends meet, being unable to quickly replace things, having to borrow money for important expenditures, untimely meeting of payments of rent and general utilities, having a creditor demanding payment, and receiving financial support from family and friends within the last month. It had a binary code– Yes and No. The index score for “Yes” ranged from 0 to 6. A higher score meant more financial problems.

To measure psychological well-being, the study used three variants of psychological well-being– mental health, self-esteem and life satisfaction, and then computed their index. Mental health measures included a 5-item Mental Health Inventory [37]. We computed an index score that ranged from 1 (never) to 6 (continuously). Respondents were asked how often they felt nervous, depressed, happy, calm or down in recent weeks. The last three items in the inventory were re-coded so that higher scores were interpreted as better mental health. Financial scarcity also has implications for self-esteem and life satisfaction, and the study measured these variables using some scales [38, 39]. While the self-esteem scale is a 10-item scale and had scores that ranged from 1 to 7, the life satisfaction scale had scores that ranged from 1 to 10. For both scales, higher scores meant higher esteem and life satisfaction. In the self-esteem inventory, items 3, 5, 8, 9 and 10 were re-coded.

Variables were analyzed at descriptive and inferential levels. Content analysis was also used to analyze the open-ended responses. Mean age, median education category and mean average monthly income were computed. The relationship between the Psychological Inventory of Financial Scarcity (PIFS) and socio-demographic variables was analysed. Regression analysis was conducted to check the association between socio-demographic variables and the PIFS.

We also examined the relationship between PIFS and financial problems and psychological well-being. We calculated the total PIFS from its four sub-components and then determined the relationship between PIFS, financial problems and psychological well-being measures. With the regression analysis (using the Ordinary Least Squares method), we tested to see if respondents who encountered more financial problems had more experience of financial scarcity, and if respondents who experienced more financial scarcity had poor psychological well-being. We further conducted a regression analysis to see if financial problems mediated between PIFS and psychological well-being. In other words, whether financial problems are positively related to PIFS which in turn is negatively related to Psychological well-being. These analyses were done using the SPSS version 26.

## Results

### Socio-demographic characteristics

As shown in Table 1 below, a total of 2237 respondents participated in this study across the six (6) geopolitical zones in Nigeria. There were more males (63.2%) than females. The mean age was 35.4 and nearly half of the respondents (49.0%) were unmarried. The greater percentage had a first degree (38.8%) and all participants were Nigerians who had resided in the country for the past 6 months that preceded the study. The greater majority were the heads of households with an average size of 5.2 household members and earned an average income of 174,723.3.

**Table 1:** Socio-demographic characteristics of respondents

Variables	Category	Frequency (*N* = 2237)	Percentage (%)
Gender	Male Female	1414 823	63.2 36.8
Age Group Mean age: 35.4	18–30 years 31–40 years 41–50 years 51 years and above	758 567 768 144	33.9 23.3 34.3 6.4
Marital Status	Single (Never Married) Married Divorced/Separated (Previously married)	1096 1047 94	49.0 46.8 4.2
Highest Level of Formal Education Attained	Primary Secondary National Diploma Higher National Diploma First Degree Master Degree Doctor of Philosophy (PhD) **Median Education: First Degree**	48 143 472 97 867 387 223	2.1 6.4 21.1 4.3 38.8 17.3 10.0
Citizenship	Nigerian Non-Nigerian	2237 0	100 -
Resident in Nigeria in the last 6 months	Yes No	2237 0	100 -
Geo-political zone	North-central North-east North-west South-east South-south South-west	251 98 100 367 875 546	11.2 4.4 4.5 16.4 39.1 24.4
If the respondent is the head of the household	Yes No	1190 1047	53.2 46.8
Household size Mean Household Size: 5.2			
If the respondent has children	Yes No	957 1280	57.2 42.8
Number of children had Mean number of children: 2.9			
Mean Average Monthly Income (In Naira -₦’000) Mean: 174,723.3 Minimum: 3,000.00 Maximum: 350,000.00	30 and below 31–90 91–150 151 − 210 211–270 271 and above	203 408 364 393 418 451	9.1 18.2 16.3 17.6 18.7 20.2

### Naira note redesign awareness and perception

The study assessed respondents’ perceptions and awareness of the redesign policy. The findings, as shown in Table 2 below, revealed that a substantial majority of the respondents (97.9%) were aware of the redesign policy, indicating widespread knowledge of its existence. Among those surveyed, 60.0% believed that the policy would be beneficial for Nigerians. However, it is noteworthy that 56.5% of the respondents expressed concerns about the timing of the policy, given the challenges associated with conducting online transactions and the limited accessibility to cash.

As a result, a significant portion of the respondents (83.0%) indicated that Nigeria is not adequately prepared for a cashless system, highlighting their perspective on the country’s readiness to transition away from cash-based transactions. These findings shed light on the mixed perceptions and concerns among the respondents regarding the benefits and challenges associated with the redesign policy, particularly in relation to the timing and Nigeria’s preparedness for a cashless system.

**Table 2:** Naira note redesign awareness and perception by respondents

Variable	Percentage (%)
Aware of the CBN policy to redesign the Naira notes	
Yes No	97.9 2.1
If the policy was in the interest of Nigerians	
Yes No	60.0 40.0
If it was a timely policy	
Yes No	43.5 56.5
If respondents used online payment platforms	
Yes No	87.4 12.6
Type of online payment platform used	
ATM Internet Banking Others POS USSD	21.1 37.2 5.9 21.1 14.7
How easy respondents’ online transactions was during the redesign policy	
Extremely Difficult Slightly Difficult Not sure Quite Easy Extremely Easy	43.4 44.8 0.0 9.8 1.9
How accessible cash was to respondents	
Extremely inaccessible Slightly inaccessible Not certain Quite accessible Extremely accessible	57.7 35.5 4.3 2.5 0.0
Nigeria is ready for a cashless economy	
Yes No	17.0 83.0

In addition to expressing concerns about the timing of the policy and the challenges associated with limited cash accessibility and online transactions, participants in the study also shared their perception that the redesign policy was politically motivated, particularly due to its timing coinciding with the preparation of the general elections. This viewpoint suggests that participants believed there may have been underlying political motives behind the implementation of the policy.

Furthermore, participants emphasized that the policy had a disproportionate impact on the masses, particularly the poor. This observation underscores the notion that individuals from lower socioeconomic backgrounds were more severely affected by the consequences of the redesign policy. These findings highlight the participants’ extensive understanding and recognition of the socioeconomic dynamics at play, which contributed to their perception of the policy as politically motivated and its adverse effects on vulnerable segments of society. Thus:

The redesign of our currency is ill-prepared, untimely, politically motivated, malicious to the common citizenry, and a vendetta. Resources are available to the few very wealthy but a scarce commodity for millions of the poor masses. From random statistics, you will see that you never find a rich man either in the bank, ATM or POS because they have the bank managers at their beck and call. However, an unprecedented traffic jam is experienced by millions of the poor, either in the bank halls (of course many banks have closed up) or at the ATM where some ignorant poor masses now keep vigil, or at the POS where the vendors now make brisk business off the poor masses. Such a fire brigade approach to the change of currency was ill-advised… Those concerned with vote buying are still those who have stiffened the flow of the lean and ill-advised printing of few Naira notes. In all, the timing is bad as it has unleashed on the poor masses untold hardship coupled with the already abused economy in our country… Fuel scarcities, epileptic power supply, exploitative internet provision coupled with the vast number of people who are internet illiterate make it more difficult to deal with. It is a pitiable situation that our leaders put the cart before the horse and play the blame game with the apex banks… and who suffers in the end? The masses… The good aim (if any) is defeated when people suffer for what they are not prepared for. No amount of talk can do justice to the mess we are in, the only thing left now is to do the needful and vote wisely. **(Male/South-West)**

Describing the unpreparedness of Nigeria for a cashless economy, participants in the study highlighted the absence of basic amenities as a major obstacle to embracing such a system. One participant aptly expressed this viewpoint:

As a nation, we don’t have any stable internet connection (the most important base for a cashless economy to thrive). The government should ensure that the internet is stable first. Also, the majority of Nigerians are largely uneducated or semi-literate hence, unbanked but, now they’re being forced to obtain bank accounts but, the larger population are still outside this category. The major obstacle to the cashless policy is very poor internet connectivity… **(Male/South-East)**.

Another stated:

The lack of basic amenities in Nigeria makes it virtually impossible to fully embrace a cashless economy. We still struggle with a consistent electricity supply, let alone access to stable internet connections. How can we expect people to rely on online transactions when they cannot even access the necessary infrastructure? It’s a significant barrier that needs to be addressed before we can truly transition to a cashless society…(**Female/Northeast**).

These statements illustrate the participant’s recognition of the essential infrastructure required for a successful transition to a cashless economy. The lack of reliable electricity and internet services in many areas of Nigeria presents a significant hindrance to the widespread adoption and effective implementation of online transactions. It underscores the challenges that must be overcome before Nigeria can fully embrace a cashless system.

The issue of inclusion was a prominent concern among participants, who expressed the view that the redesign policy and cashless system would be detrimental to vulnerable populations, such as the physically impaired and older adults. Participants noted that the absence of a support system specifically designed to accommodate the needs of these individuals would hinder their ability to thrive in a cashless economy.

One participant highlighted this concern, stating:

The rural people are already excluded as many of them do not have internet and the type of phone required for such transactions. Not only that, older adults will not be able to cope because of their poor level of digital literacy, there is no support facility for them. Those who are blind definitely will not cope. So, how will the government or the so-called system protect these groups of people and ensure their safety and participation? **(Female/North-Central)**

Recounting further, a participant said:

The policy and the cashless system seem to overlook the needs of vulnerable groups, including the physically impaired and older adults. There is currently no adequate support system in place to ensure their inclusion and participation. Without accessible platforms, tools, and assistance, these individuals may face significant barriers in carrying out transactions and navigating the cashless system. It is crucial that we consider their unique circumstances and implement measures to ensure their full integration and participation (**Female/South-west)**.

This statement reflects the participants’ recognition of the potential marginalization of vulnerable populations within the context of the cashless system. It emphasizes the need for inclusive strategies and supports mechanisms to address the specific challenges faced by the physically impaired and older adults, ensuring their equitable participation in the evolving financial landscape.

Finally, participants in the study made a connection between the redesign policy and the psychological well-being of the masses, expressing their dissatisfaction with the government’s perceived lack of concern for the psychological well-being of its citizens. This sentiment was summarized by one participant, who stated:

It is disheartening to witness how the redesign policy and its consequences have negatively impacted the well-being of the masses. The government’s apparent disregard for the mental well-being of its citizens is deeply troubling. The financial stress and uncertainty brought about by the policy have taken a toll on people’s mental health, increasing stress levels and anxiety. The government must recognize the importance of psychological well-being and take proactive measures to address the adverse effects of such policies on the mental health of its population. **(Male/South-west)**

Another expressed thus:

The policy was badly timed. I blame the government which fails to realize that some of its actions and inactions have implications for the psychological health of the masses. Take, for instance, some pictures that went viral on social media about people who got frustrated while queuing for money unsuccessfully for hours and had to go naked to the full glare of all. Another report was an old man who wept bitterly because of his inability to purchase his medication arising from his difficulty in assessing cash. Another was acts of vandalism by irate young people and the destruction of public properties. There was also a case of death recorded in a certain hospital over the inability to assess money for treatment. How can the bereaved ever get through this? These are the information I am privy to. What about others in other parts of the country that may not have been reported by the media or the majority of the people are not aware of? I feel that this policy is not advantageous in any way given the detrimental effect it has on the psychological health of the masses who suffer from this especially the poor. **(Male/South-South)**

These were reflections of the participants’ critical perspective on the government’s responsibility to consider the psychological well-being of its citizens. They highlight the negative psychological impact of the policy and emphasize the need for the government to prioritize the mental health and overall well-being of the masses in their policy-making decisions.

### Multivariate analysis of psychological inventory of financial scarcity and socio-demographic variables

In Table 3 below, we analyzed the effects of selected socio-demographic characteristics of the respondents on the psychological inventory of financial scarcity (PIFS). The coefficient of determination,$$ {R}^{2}$$ is about 0.15. It shows that about 15% of the systematic variations in the psychological inventory of financial scarcity were explained by the socio-demographic variables in the model. Similarly, the adjusted $$ {R}^{2}$$ is approximately 0.14 which implies that about 14% of the systematic variations in the psychological inventory of financial scarcity were attributed to the socio-demographic variables in the model. From the results of the tests of between-subject effects, the corrected model F-statistic ($$ F(13, 1947) = 32.08, p< 0.01$$) was significant at 1%, implying that the PIFS model was significant.

The estimated coefficient for the male gender was − 0.19 and insignificant at 5% ($$ t =-0.46, p= 0.64$$). It indicates that male respondents did not significantly experience less financial scarcity than their female counterparts during the cash crunch. On age, the estimated coefficient was positive and significant at 1% ($$ t = 8.51, p< 0.01$$), showing that older individuals experienced more financial scarcity during the cash crunch arising from the redesign policy. The estimated coefficient of individuals with secondary levels of education and above was positive and significant and implies that individuals with higher levels of education probably had more access to cash compared to those with no formal and primary education. The result, therefore, indicates that the less educated in society have less access to cash compared to the educated within the period of cash scarcity.

The estimated coefficient of the household size of respondents was significant and implied that individuals with larger households experienced more financial scarcity during the cash crunch. Although the estimated coefficient of income of respondents was positive, it was insignificant at 5%, showing that the income of individuals did not significantly determine their access to cash during the demonetization period. In other words, the level of income of individuals was not a significant determinant of how much money they were allowed to access in the banks.

**Table 3:** Psychological inventory of financial scarcity model

Dependent Variable: Psychological inventory of financial scarcity					
Variable	Variable Category	Coefficient	Std. Error	T-ratio	*P*-value
Intercept		1.490	0.279	5.34	0.000
Gender	Male	− 0.019	0.041	-0.46	0.644
	Female (R)	0	.	.	.
Age		0.024	0.003	8.51	0.000
Level of education	Secondary school certificate	0.998	0.146	6.85	0.000
	National Diploma	0.856	0.129	6.65	0.000
	Higher National Diploma	1.055	0.129	8.18	0.000
	First Degree	0.856	0.111	7.72	0.000
	Master Degree	1.013	0.117	8.63	0.000
	Doctor of Philosophy	0.685	0.124	5.52	0.000
	Others (R)	0	.	.	.
Household size		0.061	0.008	7.52	0.000
Income		0.003	0.019	0.18	0.860
Summary Statistics					
$$ {\varvec{R}}^{2}=0.15$$	$$ {\stackrel{-}{R}}^{2}=0.14$$	F-stat $$ (13, 1847) = 32.08, p < 0.01$$			

Note. R indicates the reference group or category and hence its estimated coefficient is normalized to zero

### Multivariate analysis of psychological well-being, psychological inventory of financial scarcity, and financial problems

In Table 4 below, we analyzed the effects of the Psychological Inventory of Financial Scarcity (PIFS) and its interaction with financial problems on psychological well-being. The adjusted coefficient of determination indicates that about 42% of the systematic variations in psychological well-being were explained by the predictive variables in the model. From the results of the tests of between-subject effects, the corrected model F-statistic was significant at 1% level of significance. This, therefore, implies that the psychological well-being model is significant. Furthermore, the estimated coefficient of marital status associated with divorced/separated and married in the model was negative and significant at 1%. This means that during the Nigerian cash crunch, individuals who were married, divorced or separated were less likely to experience psychological well-being in relation to those who were single (never married). Although both married and divorced/separated were less likely to experience psychological well-being, comparatively, the divorced/separated individuals were worse off compared to the married or single individuals.

On the age of respondents, the estimated coefficient was positive and significant, indicating that older individuals experienced more psychological well-being during the cash scarcity in Nigeria. Household size estimated coefficient was negative, -0.03 and significant showing that individuals with larger households experienced less psychological well-being during the cash crunch.

Additionally, the interaction between financial problems and psychological inventory of financial scarcity was significant at 1% ($$ t = 5.91, p < 0.01$$). This reveals that financial problem mediates with the psychological inventory of financial scarcity to influence the psychological well-being of individuals in Nigeria. Moreover, the estimated coefficient of the financial problem was negative, -0.48 and significant at 1% ($$ t = -9.06, p < 0.01$$). Its marginal effect on psychological well-being evaluated at the mean value of the psychological inventory of financial scarcity was negative (-0.17), depicting that financial problems reduced the psychological well-being of individuals.

Similarly, the estimated coefficient of psychological inventory of financial scarcity was negative (-0.53) and significant at 1% ($$ t = -10.54, p < 0.01$$). Its marginal effect on psychological well-being evaluated at the mean value of the financial problem index was also negative, -0.27, implying that psychological inventory of financial scarcity undermined the psychological well-being of individuals via financial problems during the cash crunch.

In addition, the estimated coefficient of individuals residing in the north-central, northeast, northwest, and southeast geo-political zones was negative and significant. This means that individuals who resided in these geo-political zones in Nigeria were more likely to suffer psychological distress compared to individuals in the southwest and south-south geo-political zones during the cash crunch. Overall, the results also revealed that among all the geopolitical zones, respondents from the northwest geo-political zone were most likely to experience psychological stress in relation to those from the other regions. This is closely followed by those who lived in the north-central, southeast, and northeast regions respectively. Individuals in the southwest were better off in terms of their psychological well-being compared to those in the other regions. Though it was observed that individuals in the south-south region were worse off than those in the south-west region, however, it was not significant.

Regarding income, the estimated coefficient was also positive but insignificant, meaning that individuals’ income did not significantly influence their psychological well-being during the cash crunch. A possible explanation for this finding is that the money of many individuals was trapped in the banks with little or no access to it during the cash scarcity period.

**Table 4:** Psychological well-being model

Dependent Variable: Psychological well-being							
Variable		Variable Category	Coefficient		Std. Error	T-ratio	*P*-value
Intercept			6.167		0.243	25.37	0.000
Marital status		Divorced/Separated	− 0.781		0.073	-10.76	0.000
		Married	− 0.321		0.040	-7.93	0.000
		Single (R)	0		.	.	.
Age			0.025		0.002	14.23	0.000
Household size			− 0.025		0.007	-3.56	0.000
Financial problem			− 0.480		0.053	-9.06	0.000
Psychological inventory of financial scarcity			− 0.529		0.050	-10.54	0.000
Financial problem* Psychological inventory of financial scarcity			0.088		0.015	5.91	0.000
Geo-political zone		Northcentral	− 0.595		0.050	-11.82	0.000
		Northeast	− 0.319		0.066	-4.81	0.000
		Northwest	− 0.613		0.089	-6.91	0.000
		Southeast	− 0.419		0.045	-9.39	0.000
		South-south	− 0.039		0.037	-1.06	0.292
		Southwest (R)	0		.	.	.
Income			0.004		0.015	0.308	0.758
Summary Statistics							
$$ {\varvec{R}}^{2}=0.42$$2	$$ {\stackrel{-}{R}}^{2}=0.418$$			F-stat $$ (13, 1947) = 35.44, p < 0.01$$			

*Note*. R indicates the reference group or category and hence its estimated coefficient is normalized to zero

## Discussion

The study focused on examining the Nigerian currency redesign policy and its impact on financial scarcity and psychological well-being. To measure financial scarcity, the study utilized the Psychological Inventory of Financial Scarcity (PIFS), which encompassed four different aspects: insufficient financial resources, lack of control over the financial situation, financial rumination and worry, and short-term focus. These aspects provide a comprehensive understanding of the range of experiences associated with financial scarcity. They capture individuals’ perceptions of their coping abilities, as well as cognitive shifts such as intrusive thoughts and a narrowed focus of attention. Psychological well-being, in this study, was assessed through measures of mental health, self-esteem, and life satisfaction. Lower levels of psychological well-being indicated poorer mental health, self-esteem, and life satisfaction. By examining the relationship between the Nigerian currency redesign policy, financial scarcity, and psychological well-being, the study aimed to shed light on the multifaceted impact of the policy on individuals’ financial and mental health experiences.

The respondents in the study perceived the Naira redesign policy as untimely, resulting in extreme difficulty and inaccessibility to cash. They highlighted that Nigeria is ill-prepared for a cashless economy due to the lack of basic amenities, including limited internet services and unstable power supply.

Regarding demographic variables and their association with financial scarcity during the cash crunch caused by the redesign policy, several findings emerged. Firstly, there was no significant difference between male and female respondents in experiencing financial scarcity, suggesting that the financial crisis was not gender biased. However, older individuals reported higher levels of financial scarcity. This could be attributed to factors such as higher unemployment rates and lower income levels among the older population.

Furthermore, the study found that individuals with higher levels of education had better access to cash compared to those with no formal education or only primary education. This disparity in access to cash during the period of cash scarcity may be linked to factors such as low financial inclusion, poor financial literacy, limited knowledge about available financial resources, difficulties in utilizing financial technologies, and uneven distribution of financial services that do not adequately cater to the needs of different demographic groups, particularly the less educated population in Nigeria [40]. These findings highlight the intersection between demographic variables, financial scarcity, and the challenges faced by different segments of society during the cash crunch caused by the redesign policy. They underscore the importance of addressing the barriers to financial inclusion and improving financial literacy to ensure equitable access to financial resources for all individuals, regardless of their educational background or age.

This study aligns with findings [41] which established the impact of financial and economic crises on psychological well-being. During the Nigerian financial crisis characterized by Naira scarcity, the study found that individuals who were married or divorced/separated (previously married) experienced higher levels of psychological distress compared to those who were single (never married). While both married and divorced/separated individuals were less likely to experience psychological well-being, the latter group fared worse in terms of their mental health. This contradicts previous studies [42, 43] that reported higher psychological distress among unmarried individuals and concluded that marriage or cohabitation provided better well-being and mental health, serving as a buffer during financial crises. When examining the relationship between household size, financial scarcity, and psychological well-being during the cash crunch, it can be inferred that larger households faced more financial challenges and experienced lower psychological well-being. In contrast, single individuals (never married) who had only themselves to support may have had fewer financial obligations, potentially contributing to their relatively better psychological well-being during the crisis. Therefore, in this study, being married or divorced/separated did not confer any advantage during the cash crunch in Nigeria; instead, it led to increased financial problems and lower psychological well-being. These findings shed light on the complex relationship between marital status, household size, financial scarcity, and psychological well-being during times of economic crisis. They suggest that the presence of dependents and financial responsibilities within married or previously married individuals may contribute to additional stress and financial burdens, leading to poorer psychological outcomes.

Although older individuals experienced higher levels of financial scarcity during the cash crunch resulting from the redesign policy, they paradoxically exhibited greater psychological well-being during the same period. One possible explanation for this finding is that with ageing comes a greater accumulation of life experience, wisdom, and resilience, enabling older individuals to adapt to adverse socioeconomic circumstances without significant detrimental effects on their psychological well-being. This finding is in line with other studies [44] which emphasized the dual role of mastery in mitigating the impact of economic hardship on the health of older individuals.

The geographical location of respondents also played a significant role in determining their likelihood of experiencing psychological distress. Specifically, individuals residing in the north-central, northeast, northwest, and southeast zones of Nigeria were found to be particularly vulnerable to poor psychological well-being. In the northern regions, the prevalence of extreme poverty exceeds the national average [45]. Moreover, in the southeastern region, the economic downturn resulting from the activities of kidnappers, unknown gunmen, and the forced shutdown of businesses and movement restrictions known as “Sit-at-home” on Mondays may have contributed significantly to the observed poor psychological well-being during the cash crunch.

These findings highlight the complex interplay between age, regional location, financial scarcity, and psychological well-being during times of economic crisis. They suggest that while older individuals may exhibit resilience in the face of financial challenges, socioeconomic context and specific regional factors can significantly impact psychological well-being.

The study found that individuals’ income level did not significantly determine their access to cash during the demonetization period. Despite variations in income, individuals were not able to access different amounts of money from the banks due to the limited availability of funds. Banks provided customers with equal fixed amounts of cash during withdrawals regardless of their deposits. Consequently, income level did not have a significant influence on individuals’ access to cash during the cash crunch caused by the demonetization policy.

Furthermore, the study revealed that individuals’ income did not significantly affect their psychological well-being during the cash crunch. This could be attributed to the fact that during a scarcity of money, the circulation of money is restricted and trapped. As a result, regardless of individuals’ income levels, there was no significant advantage in terms of psychological well-being because everyone was facing financial constraints. These findings contradict the social stress theory [46, 47] which suggests that individuals with lower income and vulnerable socioeconomic positions are more susceptible to lower psychological well-being during financial or economic crises. They also contradict other studies that have found income to be a strong protective factor against psychological distress [48–51].

The results highlight the unique nature of the Nigerian cash crunch caused by the demonetization policy, where income did not provide individuals with an advantage in terms of accessing cash or influencing their psychological well-being. The scarcity of money affected individuals across different income levels, leading to a more equitable impact on psychological well-being during the cash crunch period.

## Conclusion and recommendations

Based on the cross-sectional primary data, this study identified a significant association between the Psychological Inventory of Financial Scarcity (PIFS) and psychological well-being among residents in Nigeria during the cash crunch caused by the Naira redesign policy. It was found that financial scarcity led to financial problems, which in turn had a significant negative impact on individuals’ psychological well-being. These findings indicate that financial scarcity plays a mediating role in influencing psychological well-being among Nigerians.

Furthermore, the study observed variations in this association based on socio-demographic variables such as gender, age, marital status, education, income, and household size. These variables influenced the relationship between financial scarcity, financial problems, and psychological well-being. These findings provide valuable insights for policymakers, emphasizing the importance of adequately preparing the population for policy changes like demonetization and implementing measures to mitigate financial problems. Taking into account the psychological well-being of the population should be a crucial consideration for the government when devising and implementing economic policies.

The study also underscores the significance of considering the public health dimension of policy actions. Recognizing the impact of psychological well-being on population health, policymakers should prioritize measures that promote and protect the psychological well-being of individuals. This holistic approach ensures that policy actions not only address economic objectives but also safeguard the mental health and overall well-being of the population.

### Contribution to knowledge

Our study contributes to the limited body of research on the relationship between cash crunch (demonetization) and psychological well-being in Nigeria. To the best of our knowledge, there are few studies conducted in Nigeria that have specifically examined this topic, highlighting the scarcity of research on this subject in the African context, particularly in Nigeria. Most of the existing studies in this area have been conducted in European countries, the United States, and the United Kingdom, as evidenced by the literature [52].

By filling this gap in the literature, our study sheds light on the unique experiences and challenges faced by individuals in Nigeria during periods of cash crunch, and its impact on their psychological well-being. Through our investigation, we identified financial problems as a mediating variable in the relationship between financial scarcity and psychological well-being. This finding provides valuable insights into the underlying mechanisms through which financial scarcity affects individuals’ psychological well-being, highlighting the importance of addressing financial problems to mitigate the negative impact on psychological well-being.

Given the scarcity of studies in the Nigerian and African context, our research adds to the body of knowledge by providing a deeper understanding of the psychological implications of cash crunch and the role of financial problems as a mediator. It calls for further exploration and research in this area to enhance our understanding of the specific dynamics and challenges faced by individuals in Nigeria and other African countries during periods of financial scarcity, and to inform the development of targeted interventions and policies to support their psychological well-being.

### Limitations

While this study provides valuable insights into the relationship between cash scarcity arising from the Naira redesign policy and psychological well-being, it is important to acknowledge its limitations. One of the limitations is related to the sample selection process. Due to the use of an electronic sampling method, individuals who do not have access to the Internet or cannot communicate in the English language were excluded from participation. This may have resulted in a selection bias and limited the representativeness of the findings. Future studies should aim to incorporate diverse sampling methods to ensure a more inclusive representation of the population.

Another limitation is that the study focused on establishing the relationship between financial scarcity, financial problems, and psychological well-being, without specifically examining the types of psychological problems experienced by individuals and hospital admission arising from these problems during this period. It would be valuable for future research to explore the specific psychological disorders or symptoms associated with financial scarcity, such as suicide, psychosis, anxiety, fear, or depression. This would provide a more comprehensive understanding of the psychological impact of cash scarcity and help identify targeted interventions.

Despite these limitations, this study contributes valuable insights to the understanding of the relationship between cash scarcity, financial problems, and psychological well-being in the context of the Naira redesign policy. It highlights the need for further research to address these limitations and expand our knowledge in this important area, taking into account diverse populations, specific psychological disorders, and the impact on different sectors of the economy.

## Data Availability

The datasets used and/or analysed during the current study are available from the corresponding author upon reasonable request.
